# Comparative Space Use of Sympatric Sharks at a Remote Island in the South Pacific Ocean

**DOI:** 10.1002/ece3.71534

**Published:** 2025-06-09

**Authors:** Jordan K. Matley, Chloe N. Roberts, Thomas M. Clarke, Lauren Meyer, Michael P. Doane, Elizabeth A. Dinsdale, Mark Scott, Adam Barnett, Charlie Huveneers

**Affiliations:** ^1^ College of Science and Engineering Flinders University Adelaide South Australia Australia; ^2^ Georgia Aquarium Atlanta Georgia USA; ^3^ Norfolk Island National Park Burnt Pine Australia; ^4^ Biopixel Oceans Foundation Cairns Australia

**Keywords:** aggregation, hotspot, ISRA, provisioning, seamounts, site fidelity

## Abstract

Seamounts and offshore islands support unique assemblages of sharks. The aim of this study was to contribute to the limited knowledge of how co‐existing shark species partition spatial resources at these remote bathymetric features, particularly those influenced by a mix of temperate and tropical oceanographic conditions, as well as human activities. The space use of 34 dusky ( *Carcharhinus obscurus* ), 32 Galapagos ( *C. galapagensis* ), 47 tiger ( *Galeocerdo cuvier* ), and 20 sandbar ( *C. plumbeus* ) sharks was monitored with acoustic telemetry between 2021 and 2024 in coastal waters around Norfolk Island, Australia, a remote island in the southwestern Pacific Ocean. Seasonal patterns of residency and fine‐scale site fidelity were compared across species during this multi‐year study. Tiger sharks, consisting primarily of mature females, were seasonally present at Norfolk Island with peak residency during the Austral summer (November to April). A similar pattern existed for juvenile sandbar sharks, although departures from the island were not as common. Both species rarely overlapped in space with each other or resident (year‐round) juvenile dusky and Galapagos sharks, which were often detected in shallow waters at boating/fishing piers where incidental feeding readily occurs. Tiger sharks also showed a strong affinity to a specific area on the west side of the island where organic waste (livestock) disposal into the ocean has been ongoing for several decades. Nearshore areas of Norfolk Island support mature and immature sharks in unique ways, and human activities may further influence the space use of these species. The consistent use of specific areas around Norfolk Island across species highlights the importance of offshore islands as hotspot areas fulfilling basic biological needs at both local and oceanic scales.

## Graphical abstract

The space use of 34 dusky ( *Carcharhinus obscurus* ), 32 Galapagos ( *C. galapagensis* ), 47 tiger ( *Galeocerdo cuvier* ), and 20 sandbar ( *C. plumbeus* ) sharks was monitored with acoustic telemetry between 2021 and 2024 in coastal waters around Norfolk Island, Australia, a remote small island in the southwestern Pacific Ocean. Nearshore areas of Norfolk Island support mature and immature sharks in unique ways, and human activities may further influence space use of these species. The consistent use of specific areas around Norfolk Island across species highlights the importance of offshore islands as hotspot areas fulfilling basic biological needs at both local and oceanic scales.

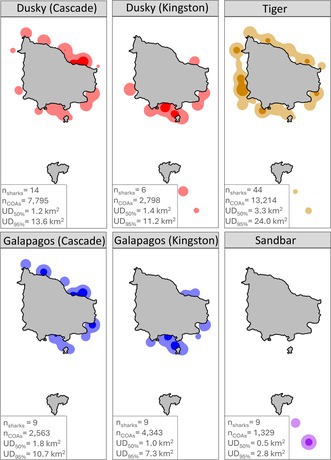

## 1 Introduction

Seamounts and offshore islands are recognized as important habitats for a range of marine species and ecosystem functions (Boehlert and Genin 1987; Wilson Jr and Kaufmann 1987). Supported by dynamic ocean currents and upwelling events (Williams et al. 2006; Morato et al. 2007), surrounding waters often have high rates of primary productivity, which in turn lead to high species richness in otherwise depauperate pelagic systems (Boehlert and Genin 1987; Morato et al. 2010). The species that are attracted to these unique and localized environments commonly use them for foraging, reproduction, development, protection, navigation, or locations to gather (Wilson Jr and Kaufmann 1987; Morato et al. 2010). Eddy formations and deep‐water surrounding seamounts and islands act as natural barriers to dispersal for marine species (Williams et al. 2006; Hirschfeld et al. 2021). Accordingly, seamounts and offshore islands often support a range of endemic species or isolated populations, resulting in unique communities distinct to specific locations (Richer de Forges et al. 2000; Williams et al. 2011).

Seamounts and offshore islands are also important ecological features shaping predator–prey relationships. For example, abundance, biomass, and diversity of predatory fishes can be several times higher at seamounts and remote islands compared to nearby low‐lying areas (Morato et al. 2008; Cresswell et al. 2023; Weber et al. 2025). Sharks are often prolific in these predator assemblages and are commonly found at remote islands and seamounts throughout the Pacific Ocean, including St. Joseph Atoll, Seychelles (Weideli et al. 2023), the Hawaiian island chain (Papastamatiou et al. 2006), the Line Islands (Sandin et al. 2008), the Cocos Islands (White et al. 2015), the Galapagos Islands (Hearn et al. 2014; Hirschfeld et al. 2023), seamounts of the Coral Sea (Barnett et al. 2012; Letessier et al. 2019), and the Revillagigedo Archipelago (Klimley, Arauz, et al. 2022), among others. Sharks have critical roles in marine ecosystems through direct and indirect predation effects (Dedman et al. 2024) and competition between sympatric shark species can further impact the role they have in ecosystems (Klinard et al. in review). Thus, evaluating the distribution of sharks and their interactions among species with similar niches provides valuable information about how their ecology has been shaped by the distinct evolutionary and biogeographic processes common to seamounts and offshore islands (Whittaker et al. 2008; Dawson 2016). Sharks commonly inhabiting offshore islands and seamounts range from those considered highly mobile, such as tiger ( *Galeocerdo cuvier* ), Galapagos ( *Carcharhinus galapagensis* ), sandbar ( *C. plumbeus* ), and scalloped hammerhead ( *Sphyrna lewini* ) sharks, to species associated with smaller home ranges and specific habitats, such as grey reef ( *C. amblyrhynchos* ), whitetip reef ( *Triaenodon obesus* ), and blacktip reef ( *C. melanopterus* ) sharks (Barnett et al. 2012; Hearn et al. 2014; Acuña‐Marrero et al. 2018). A variety of residency and site fidelity patterns are also exhibited by sharks at these hotspot areas, with some species using them seasonally as part of migrations (Jacoby et al. 2022; Matley et al. 2025) and others remaining year‐round (Barnett et al. 2012; Papastamatiou et al. 2015; Klimley, Ketchum, et al. 2022). Seamounts and offshore islands support neonates, juveniles, subadults, and adults (Wetherbee et al. 1996; Barnett et al. 2012; Acuña‐Marrero et al. 2018), providing habitats essential for parts of the entire lifecycle of a species. Consequently, some populations may be closed and at greater risk of extinction through overexploitation or low genetic diversity due to limited immigration/emigration coupled with mating (Ovenden 2013; Hirschfeld et al. 2023).

The diversity of shark species that inhabit offshore islands and seamounts has the capacity to fill a variety of ecological roles due to differences in abundances, movement regimes, habitat use, and diet across life stages (Dedman et al. 2024; Klinard et al. in review). Resource partitioning, the process by which competition alters resource use, may also play a critical role enabling similar species to coexist at relatively small scales (i.e., sympatry). It can also lead to within‐species differences; for example, resource use patterns of conspecific sharks often vary ontogenetically (Curnick et al. 2019; Crear et al. 2023). Resource partitioning can be facilitated by differences in competitive abilities, predation risks, physiological tolerances, and dominance hierarchies, which can vary within and among species (Holmgren 1995; Speed et al. 2011). These variations allow individuals and species to partition resources across temporal (e.g., diel, seasonal) and spatial (e.g., depth) scales, and in response to environmental variations (e.g., temperature, salinity, habitat selection; Heupel et al. 2018; Lea et al. 2016; Navarro et al. 2016; Papastamatiou et al. 2018). Identifying how sympatric species interact with each other and their environment is relevant to present‐day management because conservation priorities are increasingly being directed towards maintaining ecosystem function (Cinner et al. 2020; Dedman et al. 2024). Thus, identifying functional redundancies or lack thereof is an important step to help prioritize conservation efforts.

While elasmobranch assemblages and sympatry at seamount and offshore island ecosystems have been studied (e.g., Galapagos Islands—Hearn et al. 2014; Cocos Islands—White et al. 2015; Seychelles—Weideli et al. 2023; Ascension Island—Weber et al. 2025), there is a considerable paucity of knowledge relating to how habitat and resources are partitioned within and between species at these locations. Here, we investigated the space use of four different sharks at Norfolk Island, a small island ~740 km from the nearest land mass. The four species included: tiger shark—wide ranging throughout temperate and tropical waters; dusky shark ( *C. obscurus* )—highly migratory, usually found nearshore or within the continental shelf; Galapagos shark—patchy tropical/subtropical distribution, usually resident to seamounts or offshore islands; and sandbar shark—migratory within temperate and tropical coastal waters (Ebert and Fowler 2021). Our main objective was to compare seasonal patterns of residency and space use of these coexisting species during a multi‐year acoustic tracking study. Specifically, we tested whether residency and roaming indices, site preferences, and activity space overlap differed across species, monthly periods, and release locations to identify the main factors associated with the space use of sympatric sharks at Norfolk Island. Findings will contribute to the limited knowledge of how sympatric sharks partition resources at offshore islands, particularly those influenced by a mix of temperate and tropical oceanographic conditions.

## 2 Methods

### 2.1 Study Area

**FIGURE 1:**
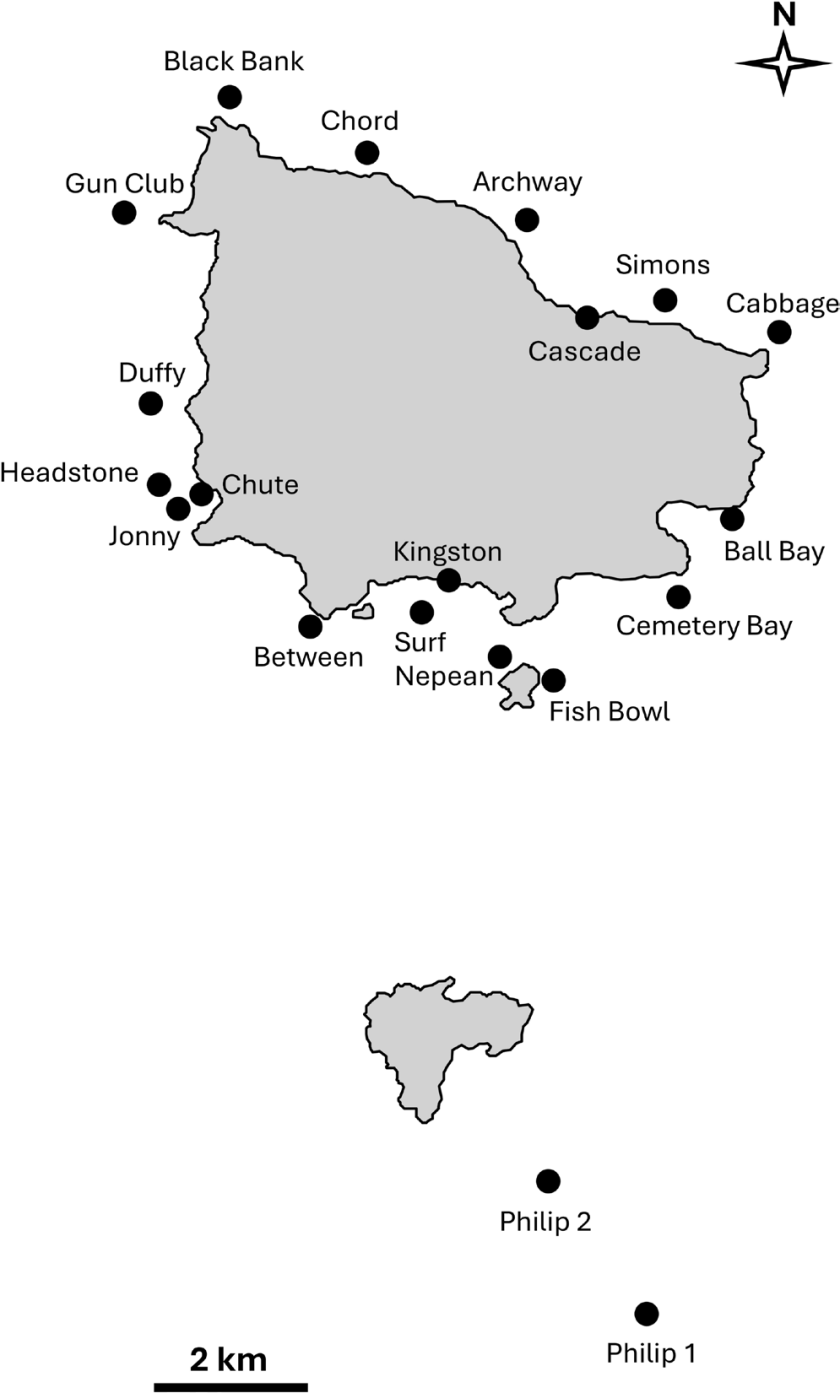
Study area at Norfolk Island (top island), Nepean Island (island between Nepean and Fish bowl), and Phillip Island (bottom island). Black dots and associated identifiers indicate receiver locations.

Norfolk Island (29°02′05.0″S, 167°57′23.7″ E; Figure 1) is a small (35.7 km^2^), inhabited (population of 2188 as of 2021 census) Pacific Ocean island, ~1400 km from the east coast of Australia and ~740 km northwest of New Zealand. It lies within the Norfolk Ridge, an oceanic ridge system between 1000 and 2000 m deep interspersed with seamounts and islands that extends north to New Caledonia and south to New Zealand. The Norfolk Ridge is an Important Shark and Ray Area (ISRA) providing connectivity for large tiger sharks to/from Norfolk Island during seasonal migrations in the western South Pacific Ocean (IUCN SSC Shark Specialist Group, 2024a; Matley et al. 2025). Norfolk Island is also an ISRA, supporting a significant feeding area (tiger sharks), a threatened species (dusky sharks), and an undefined aggregation (Galapagos sharks; IUCN SSC Shark Specialist Group, 2024b). Two smaller uninhabited islands are 1 and 6 km to the south of Norfolk Island, Nepean Island and Phillip Island, respectively. Sea surface temperature at Norfolk Island ranges from 18°C to 26°C—the coldest waters during the Austral winter (June—August) and warmest during the Austral summer (December—February; Pendoley and Christian 2012). Norfolk Island is located at the southern limit of the Tropical Convergence, an area where alternating influences of tropical and temperate currents create an unusual mix of marine fauna and flora, some of which are endemic or rare elsewhere (Francis 1993). More than a dozen species of elasmobranchs have been documented around the island (Francis 1993). Norfolk Island overlaps with the Norfolk Marine Park, the West Norfolk Ridge Ecologically or Biologically Significant Marine Area (EBSA; Convention the Biological Diversity 2024), and the Norfolk Island/Phillip Island Key Biodiversity Area (Key Biodiveristy Areas 2024).

### 2.2 Receiver Array

An array of 18 acoustic receivers (13 VR2Ws, 5 VR2ARs, innovasea.com) was deployed around Norfolk Island in February 2021 (Figure 1), with servicing and data downloads annually between February and April. An additional two receivers were deployed south of Phillip Island and one receiver east of Nepean Island (Fish bowl) in February 2023. Receivers were strategically positioned to encompass movements of sharks along the coast of the entire island, with additional sites selected due to local knowledge of notable habitat or shark abundance (e.g., popular diving sites: Fish bowl, Jonny's Stone (hereafter referred to as Jonny); fishing sites: Phillip Island; tiger shark aggregation area: Headstone). Additionally, Kingston and Cascade sites were located directly next to fishing/boating piers where sharks are often observed. Each receiver was deployed ~1 m off the seafloor either by attaching it to a steel fence post hammered into the substratum, affixing it to pier pylons, or mooring it to a chain anchor (and subsurface float) with an acoustic release mechanism. Deployment depth was between a few metres (i.e., at the piers) and 50 m (i.e., Phillip Island), but most were 18–25 m deep. Detection range, while variable, was conservatively estimated to be ~250 m (50% detection efficiency; Figure S1). Detection data were included up to the receiver download in April 2024.

### 2.3 Shark Tagging

Between February 2020–2023, 34 dusky, 32 Galapagos, 47 tiger, and 20 sandbar sharks were caught and tagged at Norfolk Island (Table 1). Shark capture and tagging varied between target species. Dusky and Galapagos sharks, tagged at Kingston and Cascade piers, were caught via a handline consisting of an 8–10 mm polypropylene/polyethylene blend rope, and landed on the lower steps of the pier for tagging. Sandbar sharks were caught using rod‐and‐reel from a vessel and brought on board for tagging. Tiger sharks were caught via free‐floating baited drumlines consisting of a vertically orientated polypropylene/polyethylene mainline (8–10 mm) and a ~1.5 m 500 lb. wire trace leader attached to a hook. A large polyform buoy was attached to the top of the mainline, with one smaller buoy connected 1.5–2 m from the main buoy. Drumlines were retrieved immediately after the main surface float became submerged, indicating capture, and tiger sharks were secured to the side of the vessel for tagging. All sharks were caught using circle hooks (size 13/0–16/0). Once landed and secured, sharks were inverted with a continuous flow of water irrigating the gills, and surgical procedures were carried out to insert a V16 acoustic transmitter (Innovasea; random interval of 80–140 s for tiger, dusky, and Galapagos sharks; 90–150 s for sandbar sharks; 3650 days expected battery life) into the peritoneal cavity. Tagging procedures followed standard international protocols (Holmes et al. 2022) consisting of making a ~4 cm ventral incision, inserting the transmitter, and sealing the incision with either interrupted cruciate or simple interrupted suture techniques using Gauge 2 Polyglycolic Acid absorbable sutures (silverglide.com.au). Following acoustic tagging, individuals were also tagged with an external identification tag (hallprint.com) in the dorsal musculature at the base of the first dorsal fin to avoid double‐tagging if recaptured. Sharks were measured (i.e., total length), sex was recorded, and maturity was identified based on species‐specific length‐at‐maturity estimates (Simpfendorfer 1992; Wetherbee et al. 1996; Whitney and Crow 2007; Geraghty et al. 2015; Holmes et al. 2015). The tagging process (i.e., from securing to release) took ~4 min.

**TABLE 1:** Tagging summary of sharks at Norfolk Island.

Species	First tagged	Number tagged	Number analyzed	Female (immature: mature)	Male (immature: mature)	Total length (m; min)	Total length (m; max)	Total length (m; mean ± standard error)	Tagging location			
									Cascade	Kingston	Phillip Island	Headstone
Dusky	Feb‐2021	34	20	13 (13:0)	7 (6:1)	1.65	2.78	2.28 ± 0.06	14	6	0	0
Galapagos	Feb‐2021	32	18	13 (13:0)	5 (5:0)	1.34	1.97	1.64 ± 0.05	9	9	0	0
Sandbar	Feb‐2023	20	9	6 (6:0)	3 (3:0)	1.25	1.49	1.38 ± 0.03	0	0	9	0
Tiger	Feb‐2020	47	44	39 (2:37)	5 (1:4)	2.34	4.36	3.83 ± 0.06	0	0	0	44

*Note:* Summary information (apart from number tagged) is based on individuals detected for a period > 14 days (i.e., those used in analyses).

Activities involving handling and tagging sharks were approved under the Australian Government's “Environment Protection and Biodiversity Conservation Regulations 2000” and “Access to Biological Resources in a Commonwealth Area for Non‐Commercial Purposes” issued to Dr. Lauren Meyer (Flinders University) under Permit number AU‐COM2021‐503. All work conducted with animals was approved by James Cook University Animal Ethics Committee (A2864).

### 2.4 Analysis

#### 2.4.1 Preliminary Filtering

Acoustic detections of all tagged individuals underwent preliminary data exploration to remove any data points that were reflective of a shed tag or an animal that died by filtering out consecutive detections at a single receiver for a period of multiple days (Klinard and Matley 2020). The first 48 h after release were not included in the analysis to reduce the potential for quantifying behavior resulting from the tagging event (Papastamatiou et al. 2009). Additionally, individuals that had a detection period (i.e., number of days between the 48‐h period after release and date of last detection) of 2 weeks or less were not included in the analysis because these animals likely left the study area and were not representative of coastal habitat use around Norfolk Island.

#### 2.4.2 Residency and Roaming

A residency index (ResI) was used to identify the relative presence of each shark around Norfolk Island during their detection periods. It was calculated as the number of days with at least one detection (at any receiver site) divided by the number of days during the detection period (from tagging date to last detection; Matley et al. 2016). The detection period was selected to quantify residency (as opposed to the tag life or study period) because it was not possible to know the fate of the animal following the last detection.

A roaming index (RoI) was used to measure the relative mobility of each shark around Norfolk Island. It was calculated as the number of unique receiver sites with detections divided by the total number of receiver sites available (Matley et al. 2016). Some receiver deployments varied in time during the study (see Section 2.1); therefore, only receivers that were present during each animal's detection period were considered when calculating individual roaming indices.

Residency and roaming were also calculated at monthly intervals. Months at the start and end of a detection period were not included unless they incorporated > 14 days to avoid estimating indices from small sample sizes—exploratory sensitivity tests indicated that index values typically stabilized after a 14‐day period each month. A generalized additive mixed‐effect model (GAMM) approach, using the gamm4 R package (Wood et al. 2017), was used to explore differences in residency and roaming among months and species (e.g., *s(Month, by = Species)*). A non‐linear cyclic (bs = “cc”) smoother was placed on the month variable (*k* = 12), with animal/transmitter ID and year as random variables. Smooth terms (i.e., month by species) with *p*‐values < 0.05, based on the restricted maximum likelihood (REML) approach, were considered significantly non‐linear (i.e., slope ≠ 0).

#### 2.4.3 Activity Space

Activity space, defined here as the main areas that were used within the receiver array at Norfolk Island, was quantified for each species using kernel density estimation within the adehabitatHR R package (Calenge 2006). To avoid biasing estimates to the western side of the island where three receiver sites were deployed within a few 100 m of each other (i.e., Headstone, Chute, and Jonny; Figure 1), we only incorporated detections at Headstone (the site deployed the longest). Centers of activity (COAs; Simpfendorfer et al. 2002) at 2‐h intervals were used as individual locations instead of raw detections to help account for movements between receivers. Stochasticity was incorporated into COAs by randomly relocating raw detections ±0–25 m away from the receiver location. Any COA location that fell on land was reassigned to the nearest shore area and land was not incorporated in activity space estimates. A smoothing parameter (*h*) of 300 was used to estimate 50% and 95% utilisation distributions (UDs) based on successive sensitivity explorations to match UD size estimates (at one receiver) with predicted detection ranges. Given the contiguous (and accessible) nature of the shoreline, as well as the constrained positioning and kernel smoothing approaches, we deemed it unnecessary to use a kernel UD estimator specifically incorporating complex coastlines (e.g., Barry and McIntyre 2011).

Overlap between each species' activity space, quantified as the area of overlap between two species relative to the mean UD size of each species, was also calculated to compare high use areas. Differences in activity space overlap among dusky and Galapagos sharks tagged at different sites (i.e., Cascade and Kingston sites) were also quantified to evaluate whether space use patterns were primarily species‐ or location‐~25 driven.

#### 2.4.4 Site Differences

Site preference around Norfolk Island was evaluated using a permutational multivariate analysis of variance (PERMANOVA) within the vegan R package (Bray‐Curtis distance matrix; 999 permutations, Dixon 2003), testing whether the relative use of each receiver site differed across species, month, and tagging location, as well as the biological variables sex and size (and each factor's interaction with species). Data exploration showed that tiger sharks consistently used different areas of Norfolk Island compared to the other species. Additionally, > 99% of sandbar shark detections were from Phillip Island. Therefore, separate PERMANOVAs were conducted for tiger sharks (without tagging location as a fixed effect since there was only one tagging location) and dusky and Galapagos sharks, while sandbar sharks (all of which were likely immature at < 1.5 m TL; Geraghty et al. 2015) were not analyzed further. The tagged dusky (< 2.7 m total length; TL) and Galapagos (< 2.0 m TL) sharks were likely all immature except one male dusky shark (2.8 m TL; Table 1; Wetherbee et al. 1996; Geraghty et al. 2015); therefore, size categories for both species were selected in a mechanistic approach based on identifying the largest gap in TL among individuals (within the 25th to 75th percentile of all sizes for that species). The resulting size cut‐offs (i.e., small vs. large) were 2.30 m TL and 1.54 m TL for dusky and Galapagos, respectively. The size categories of tiger sharks were based on expected size‐at‐maturity estimates (Simpfendorfer 1992; Whitney and Crow 2007; Holmes et al. 2015), in which individuals < 3.0 m TL were considered immature (two females and a male) and all others were mature (Table 1). A few receiver sites were not included in this analysis (i.e., Cabbage, Fish bowl, Phillip1, and Phillip2) because they were not deployed for the entirety of the study, and, as above, the Chute and Jonny sites were excluded since they were located near the Headstone site. *p*‐values < 0.05 among fixed factors (and interaction terms) were considered to significantly differ in relation to the proportional use of receiver sites, but *R* ^2^ values were used to evaluate the relative explanatory capacity of each significant relationship.

## 3 Results

A total of 20 dusky (mean TL ± standard error: 2.28 ± 0.06 m), 18 Galapagos (1.64 ± 0.05 m), 9 sandbar (1.38 ± 0.03 m), and 44 tiger (3.83 ± 0.06 m) sharks were analyzed (Table 1). Seven sharks (dusky: *n* = 1; Galapagos: *n* = 4; sandbar: *n* = 1; tiger: *n* = 1) were not analyzed because they were detected for 2 weeks or less, and 28 sharks were never detected (dusky: *n* = 7; Galapagos: *n* = 9; sandbar: *n* = 10; tiger: *n* = 2). Additionally, detections from one dusky shark were removed completely because its transmitter was consistently detected on only one receiver for over a year, resembling a shed tag or dead animal. The mean detection periods of the analyzed sharks were 498 ± 49 days (dusky), 471 ± 73 days (Galapagos), 313 ± 47 days (sandbar), and 732 ± 69 days (tiger; Table 2).

**TABLE 2:** Detection summary of sharks at Norfolk Island.

Species	Days detected (min)	Days detected (max)	Days detected (mean ± SE)	Detection period (min)	Detection period (max)	Detection period (mean ± SE)	Residency (min)	Residency (max)	Residency (mean ± SE)	Roaming (min)	Roaming (max)	Roaming (mean ± SE)
Dusky	23	409	225 ± 26	89	1134	498 ± 49	0.10	0.71	0.45 ± 0.04	0.17	1	0.67 ± 0.04
Galapagos	25	631	198 ± 42	62	1141	471 ± 73	0.15	0.67	0.41 ± 0.04	0.07	0.83	0.52 ± 0.06
Sandbar	5	243	79 ± 27	27	413	313 ± 47	0.02	0.59	0.23 ± 0.06	0.06	0.17	0.10 ± 0.01
Tiger	2	403	134 ± 17	55	1514	732 ± 69	0.01	0.84	0.24 ± 0.03	0.13	0.95	0.71 ± 0.03

*Note:* Only individuals detected for a period > 14 days were included.

### 3.1 Residency and Roaming

**FIGURE 2:**
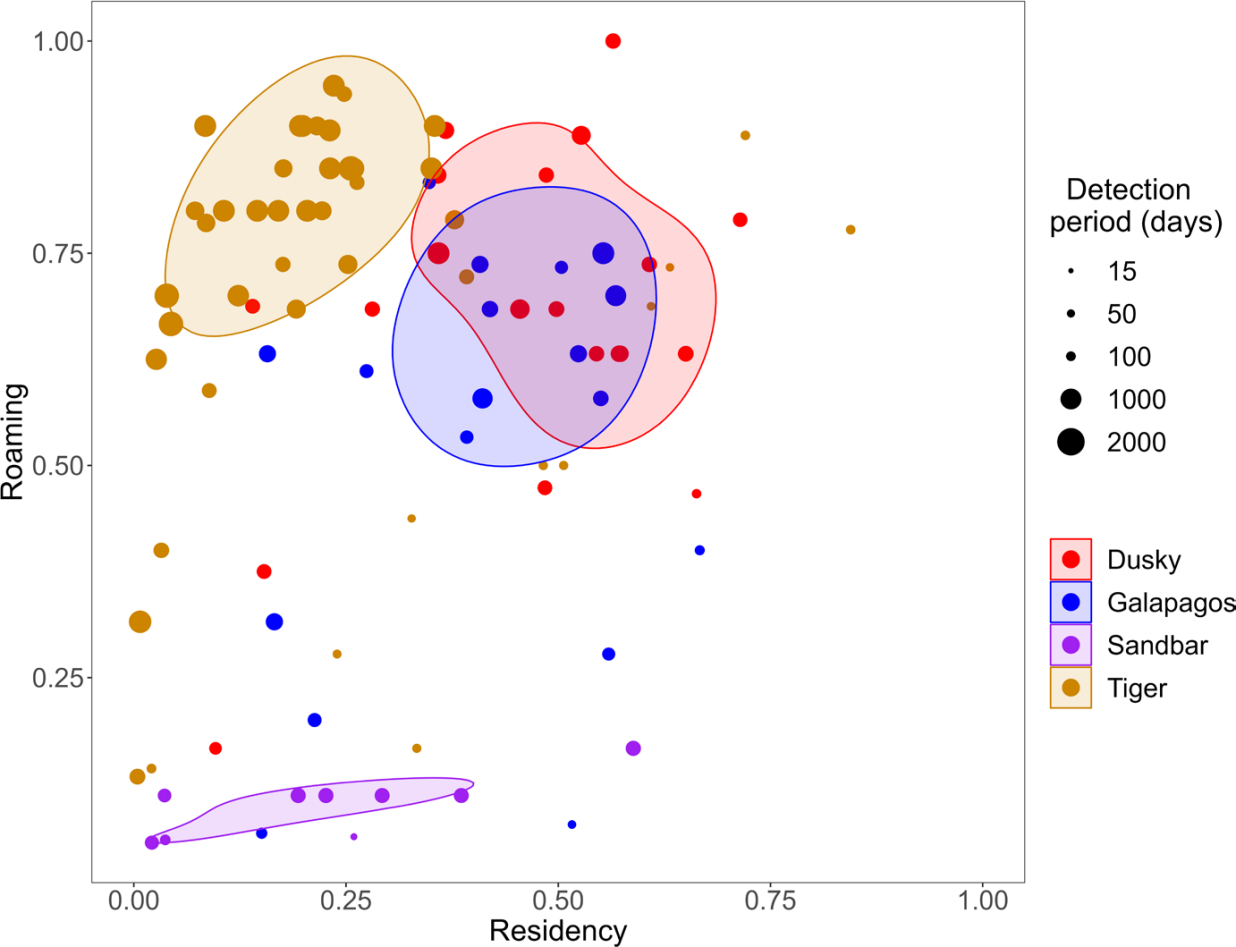
Residency and roaming indices for corresponding individuals across species. The colored ellipses represent 50% utilization distributions (from kernel density estimation) to highlight broad roaming‐residency patterns. The size of points reflects the detection period of each individual.

Dusky and Galapagos sharks had high residency and roaming indices (dusky shark: ResI = 0.45 ± 0.04, RoI = 0.67 ± 0.04; Galapagos shark: ResI = 0.41 ± 0.04, RoI = 0.52 ± 0.06; Table 2; Figure 2). The residency index of tiger sharks was lower (0.24 ± 0.03) because they seasonally left the array for considerable periods, but when present, were highly mobile (roaming index: 0.71 ± 0.03). Meanwhile, sandbar sharks also had relatively low residency (0.23 ± 0.06) and very low mobility within the receiver array (roaming index: 0.10 ± 0.01) because they were almost exclusively detected at the Phillip Island sites.

**FIGURE 3:**
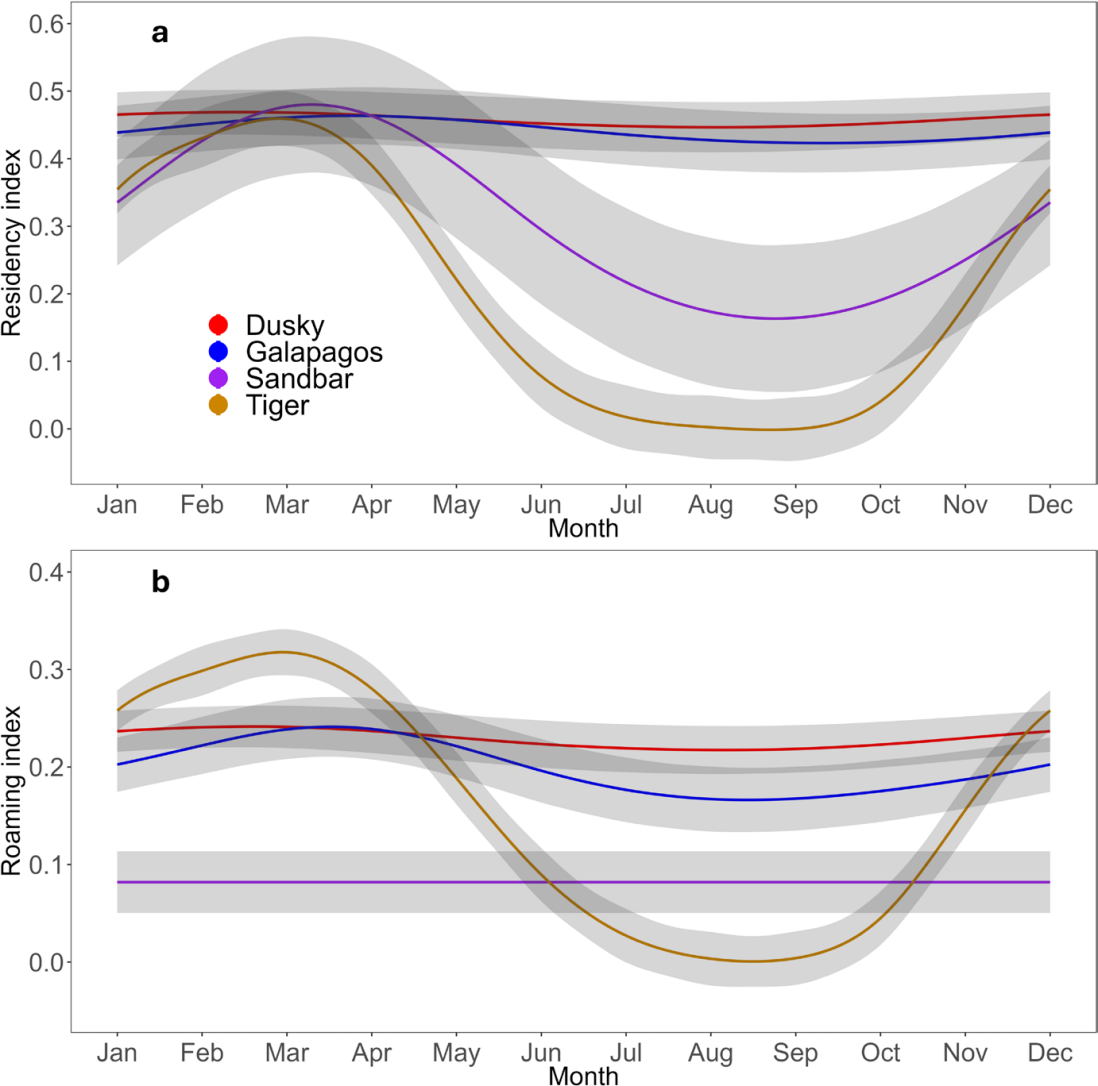
Monthly (mean ± 95% confidence interval) residency (a) and roaming (b) indices across species (all years combined) fitted using a non‐linear smoother (i.e., generalised additive mixed‐effect model).

Residency varied across months in sandbar and tiger sharks (*p*‐values < 0.05, Figure 3a; Table S1), with the residency of both species decreasing between June and October. The absence was more pronounced in tiger sharks and closely approached a residency of 0. Roaming, on the other hand, varied across months in Galapagos and tiger sharks (*p*‐values < 0.05; Figure 3b; Table S1). Roaming was highest in Galapagos sharks between March and May, while tiger shark roaming decreased during their periods of low residency (Figure 3b), reflecting their absence from Norfolk Island (as opposed to a behavioral component).

### 3.2 Activity Space

**FIGURE 4:**
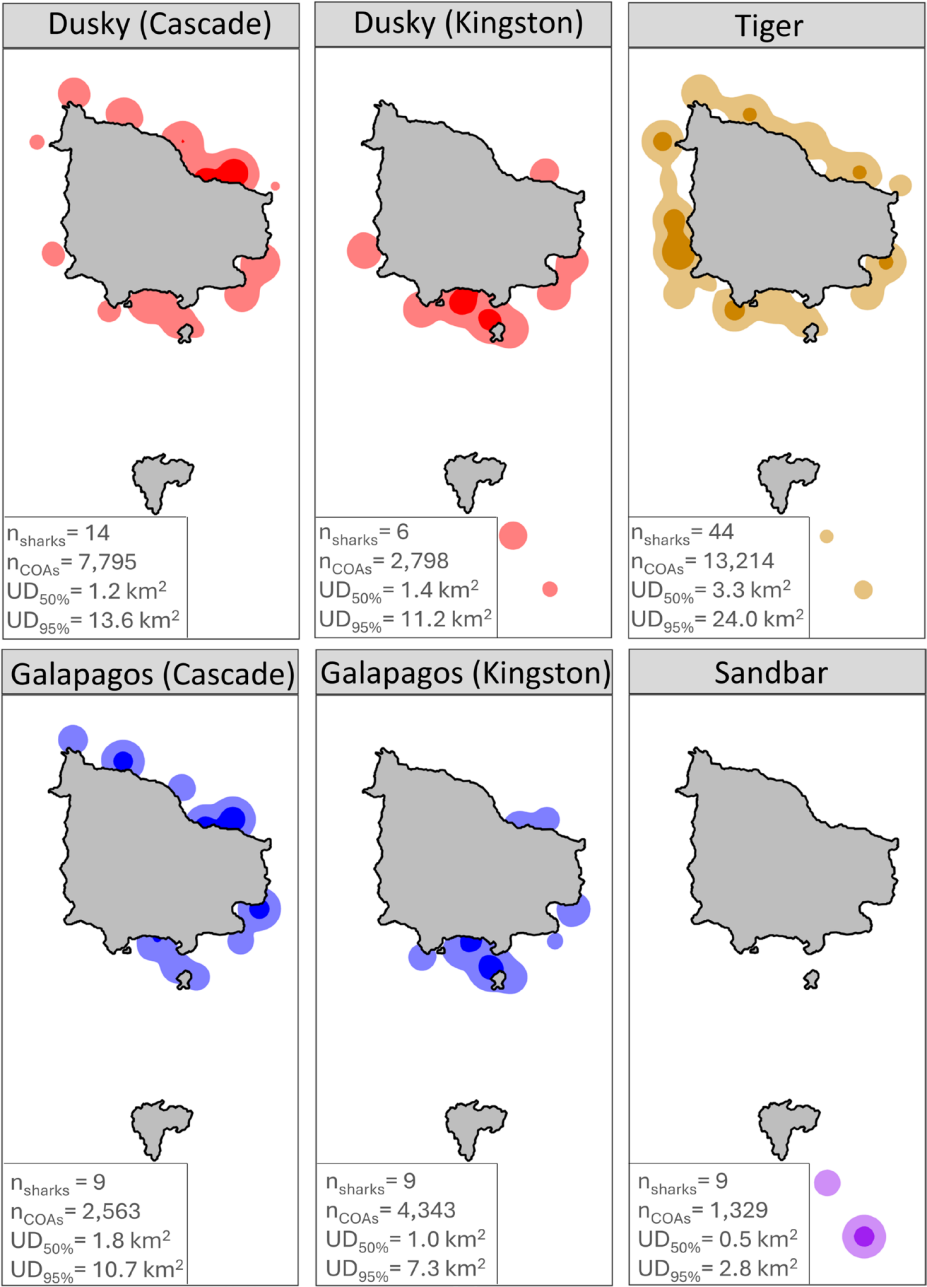
Activity spaces (50% UDs: Light green; 95% UDs: Dark green) of sharks at Norfolk Island based on 2‐h center of activity (COA) locations. Separate activity spaces were quantified for dusky and Galapagos sharks tagged at Cascade and Kingston piers.

Tiger sharks had the highest activity space around Norfolk Island (95% UD: 23.4 km^2^), followed by dusky (95% UD: 15.1 km^2^), Galapagos (95% UD: 10.7 km^2^), and sandbar sharks (95% UD: 2.8 km^2^; Figure 4). Core use areas (i.e., 50% UDs) were centered at several sites around the island for tiger sharks, while fewer sites were focal for the other species, which included areas northeast and south for the dusky and Galapagos sharks, and sites southeast of Phillip Island for sandbar sharks (Figure 4). It was evident that the tagging location of dusky and Galapagos sharks affected their activity space with more localized detections near their release site (Figure 4). Furthermore, overlap among 50% and 95% UDs was highest between dusky and Galapagos sharks at their respective tagging locations as opposed to within‐species (Figure 4; Figure S2).

### 3.3 Site Differences

**FIGURE 5:**
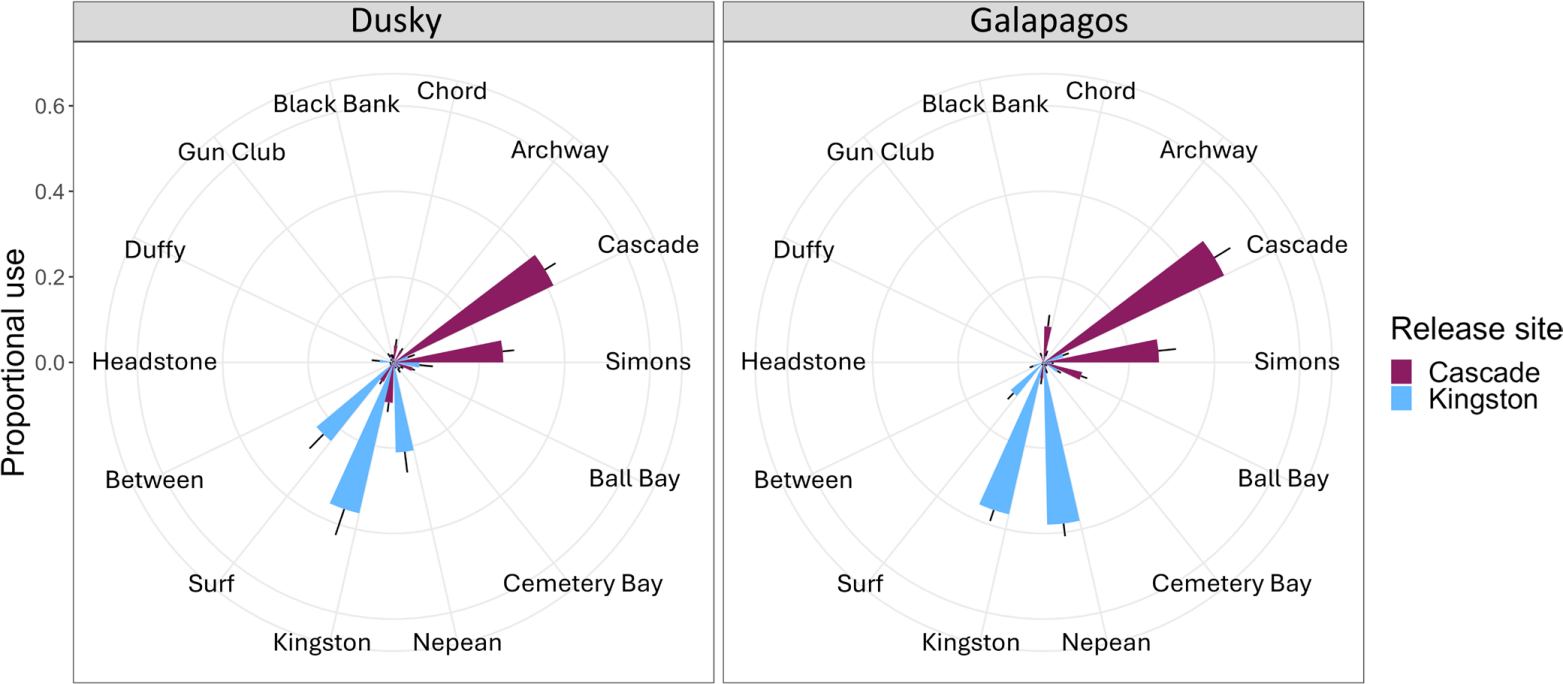
Mean (±standard error) proportion of individual hourly detections across sites for dusky and Galapagos sharks, colored by release location. Only receiver sites that were deployed for comparable time periods were included in this analysis (i.e., Cabbage, Phillip1, Phillip2, Fish bowl, Jonny, and Chute sites were not included). Note that, in addition to tagging/release location, species, month, size, and sex were significant; however, they were not plotted separately because *R* ^2^ values were low (*R* ^2^ < 0.07).

Proportional use of receiver sites varied across species, month, tagging location, size, and sex for dusky and Galapagos sharks (Table S2). However, no interaction among fixed effects was significant, and only the tagging location (*R* ^2^ = 0.29) had an *R* ^2^ value > 0.07, indicating that the other variables contributed marginally to the model. Similar to the activity space overlap, dusky and Galapagos sharks tagged at Cascade pier were mainly detected nearby at the Cascade and Simons receiver sites, whereas sharks tagged at Kingston pier were mainly detected at the Kingston, Surf, and Nepean receiver sites (Figure 5).

**FIGURE 6:**
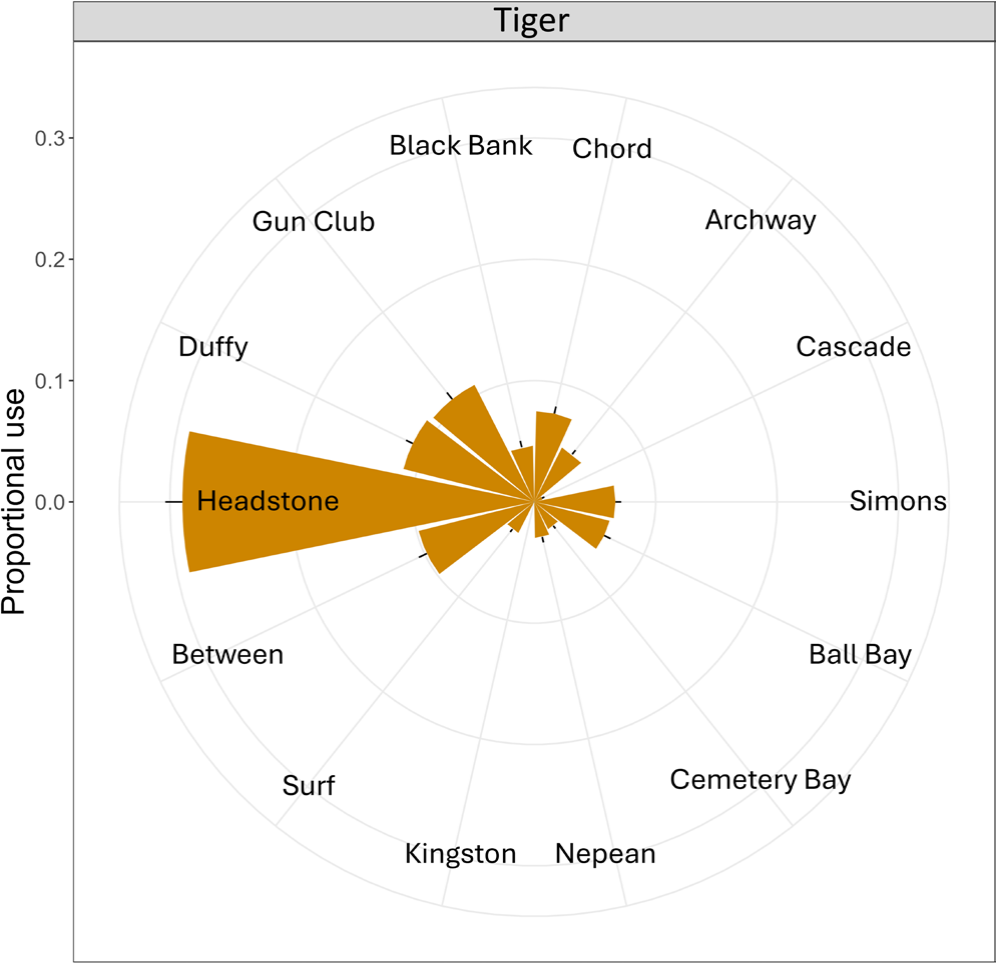
Mean (±standard error) proportion of individual hourly detections across sites for tiger sharks. Only receiver sites that were deployed for comparable time periods were included in this analysis (i.e., Cabbage, Phillip1, Phillip2, Fish bowl, Jonny, and Chute sites were not included). Note that sex, size, month, and the interaction of sex and size were significant; however, they were not plotted separately because *R* ^2^ values were low (*R* ^2^ < 0.09).

Proportional use of receiver sites also varied across sex, size, month, and the interaction of sex and size for tiger sharks (Table S3), but all *R* ^2^ values were < 0.09, once again highlighting the limited capacity to explain any differences. Overall, tiger sharks were ~3 times more likely to be detected at the Headstone receiver site than at any other site (Figure 6). Given the absence of tiger sharks seasonally (e.g., residency values were close to zero between June and October), we also explored whether dusky or Galapagos sharks used the Headstone site more during those months. In some months when tiger sharks were absent (e.g., July and August), the mean number of dusky shark detections (per individual) appeared to increase, but it was highly variable and also occurred during some months when tiger sharks were present (e.g., January; Figure S3).

## 4 Discussion

Productive coastal marine waters consist of only a small proportion of the ocean but support a substantial amount of marine life (Ramírez et al. 2017). Isolated seamounts and small islands form unique ocean hotspots by harnessing terrestrial (e.g., nutrient deposition) and marine (e.g., upwelling, photic habitat) productivity in localized areas (Boehlert and Genin 1987; Morato et al. 2010; Graham et al. 2018). The high rates of primary productivity and terrestrial input of nutrients and prey (e.g., seabirds), combined with limited human activity, typical of many seamounts and offshore islands, support dynamic assemblages of predators, such as sharks. This study characterized space use of four shark species that co‐occur around Norfolk Island, a small remote island in the South Pacific Ocean. Despite a relatively small coastal perimeter (i.e., ~25 km), there was a clear distinction in areas used among the different species. Tiger sharks used all monitored areas around Norfolk Island, whereas sandbar sharks were only detected in deep offshore waters, and dusky and Galapagos sharks preferred a few specific nearshore locations. Although several dusky and Galapagos sharks were never detected after being tagged, animals with detections were more resident to Norfolk Island and overlapped in space more than any other species pairing, with localized movements primarily near tagging locations for both species. Overall, the diversity in space use across these species provides new insight into how distinct species spatially interact around important offshore islands.

The South Pacific Ocean is largely void of significant land masses (apart from continental regions) to initiate upwelling; thus, remote islands such as Norfolk Island provide valuable resource pools for sharks that are otherwise surrounded by low productivity environments. Norfolk Island is important for mature female tiger sharks, which consistently return each year following seasonal migrations, primarily to/from New Caledonia (Matley et al. 2025). The stark absence of tiger sharks between May and October in this study attests to these return migrations. Sandbar sharks also displayed reduced residency during similar periods as tiger sharks, albeit to a lesser extent. The observed decrease in residency at Norfolk Island following summer also aligned with the seasonal timing of migrations in juvenile sandbar sharks in the western Atlantic Ocean, which were associated with seasonal shifts in photoperiod and temperature (Grubbs et al. 2007). Whether these individuals undertook seasonally mediated large‐scale migrations away from Norfolk Island or simply shifted habitats elsewhere in the area is not known due to lack of detections anywhere other than near Phillip Island. The known distribution of sandbar sharks in the Pacific Ocean is limited to coastal tropical and subtropical waters with limited evidence of movement across ocean basins (Barnes et al. 2016; Rigby et al. 2021). While juvenile sandbar sharks (smaller than those in this study) have the capacity to move up to 2800 km along continental margins (Grubbs et al. 2007), the large distances across deep oceans between Norfolk Island and other land masses may pose barriers to dispersal away from the island (Riginos and Liggins 2013; Hirschfeld et al. 2021). All sandbar sharks tagged in this study were likely immature (Geraghty et al. 2015), suggesting differences exist in space use across age or size classes. Ontogenetic discrimination at large spatial scales was demonstrated in Western Australia, where juvenile sandbar sharks were primarily caught in waters south of 26° S and adults in waters north of 26° S (Mcauley et al. 2007). The transport of warm water from the tropics to temperate latitudes via the Leeuwin Current was hypothesized to facilitate such a segregation, potentially reducing intraspecific predation and competition. Similar oceanographic patterns affect Norfolk Island, which receives tropical input from the Coral Sea via the East Australian Current and South Equatorial Current (Francis 1993). Therefore, there may also be ontogenetic shifts in space use, either locally or more broadly, that are worth exploring further. The deep habitat (~50 m; compared to other receiver sites) where sandbar sharks were captured/detected was also more aligned with findings in Western Australia where peak abundance of juveniles was in offshore nursery areas with depths between 80 and 130 m (Mcauley et al. 2007), in contrast to shallow nearshore use of estuaries and embayments in the western North Atlantic Ocean (Merson and Pratt 2001; Collatos et al. 2020) and the Gulf of Mexico (Carlson 1999). Distinct habitat use across populations, despite similar life stages, highlights the role of context‐specific factors influencing behavior (Lubitz et al. 2022).

The dusky and Galapagos sharks were the most resident species at Norfolk Island and surrounding areas without any significant seasonal variation. Individuals were detected nearly once every 2 days (i.e., ~50% residency index), indicating they remained in (or returned to) nearshore waters of Norfolk Island consistently. Like the sandbar sharks, the high proportion of immature individuals suggests size/maturity associated differences in space use, at least in relation to the nearshore areas where they were caught. The considerable overlap in space use between dusky and Galapagos sharks offers additional insight regarding resource use and behavioral patterns of two species that rarely co‐occur given their typically distinct habitat preferences (e.g., dusky sharks—inshore to offshore along continental margins and Galapagos sharks—offshore islands and seamounts). Both species were resident to their tagging location, which was a better predictor of space use than species differences. Given the spatial (this study), biological (Ebert and Fowler 2021), and dietary (Matley et al. in review) similarities, there is potential that both species fill similar ecological roles at Norfolk Island, but more research is needed, particularly regarding adult resource use. Klimley, Ketchum, et al. (2022) contrasted spatial and temporal partitioning among one dusky, six Galapagos, three silvertip, and two whitetip sharks at the Revillagigedo Archipelago using acoustic telemetry, and found that dusky and Galapagos sharks were more similar (e.g., nocturnal activity and residency) than the other species.

Most studies tracking the distribution or movement of dusky sharks have been located along continental coasts, where large‐scale seasonal migrations are common globally, including in Australia (Bartes et al. 2021; Huveneers et al. 2021), South Africa (Hussey et al. 2009), Western Atlantic Ocean (Kohler et al. 1998; Bangley et al. 2020), and Gulf of Mexico (Hoffmayer et al. 2014). However, research is limited at remote offshore islands; thus, the reliance on resources at Norfolk Island and associated movements, at least for larger immature individuals, may be distinct. For example, at the Revillagigedo Archipelago, consisting of four small volcanic islands > 350 km from mainland Mexico, one acoustically tagged dusky shark (female, 300 cm TL) showed highly localized movements and was consistently detected (RI: 0.73) on the western side of one island for almost 900 days (Klimley, Ketchum, et al. 2022). Immature dusky sharks appear to be less transient than mature individuals within continental margins, albeit still highly mobile; for example, in Western Australia smaller individuals were only detected at receivers located between 31° S and 35° S compared to larger individuals which commonly showed displacements between 22° S and 35° S (Braccini et al. 2018) and across geopolitical management jurisdictions (Rogers et al. 2013; Huveneers et al. 2021). Braccini et al. (2018) also estimated that large‐scale migrations began when females and males reached 222 and 212 cm (fork length), respectively. Thirteen of the 20 dusky sharks at Norfolk Island were above these proposed size cut‐offs, but no size‐related difference was identified.

The propensity for Galapagos sharks to inhabit oceanic islands in the South Pacific Ocean, such as Norfolk Island, is well documented (Heagney et al. 2007; Duffy 2016; Acuña‐Marrero et al. 2018; Mitchell et al. 2024). Often, they are the only shark species observed at the locations (A. Barnett, pers. obs.) and can be genetically isolated from other conspecific populations (van Herwerden et al. 2008), highlighting their unique affinity to distinct seamounts and oceanic islands. Like other locations (Galapagos Islands—Acuña‐Marrero et al. 2018; Easter Island—Morales et al. 2021; Lord Howe Island—Mitchell et al. 2024; Ascension Island—Weber et al. 2025), immature Galapagos sharks at Norfolk Island were highly resident and detected in nearshore areas, while adults were never captured. At one of the nearest islands to Norfolk Island—Lord Howe Island (~900 km away; with similar bathymetric features)—the majority of individuals in fished waters along the islands' shelves (< 100 m) were juveniles (Mitchell et al. 2024). It is surmised that adults used deeper habitats or undertook seasonal migrations elsewhere (Wetherbee et al. 1996; Meyer et al. 2010; Hearn et al. 2014).

While Norfolk Island, as a whole, acts as an ocean oasis, specific areas of Norfolk Island were also important for tiger, dusky, and Galapagos sharks. Tiger sharks were mostly detected on the west side of the island, particularly at the Headstone receiver site. The waters around Norfolk Island are very dynamic due to its location near the southern limit of the Tropical Convergence and within the Norfolk Eddy, with ocean currents commonly moving to Norfolk Island from the west (Francis 1993; Williams et al. 2006). Thus, prey may be more abundant on the west side of Norfolk Island. There is also a seasonal nesting/breeding colony of wedge‐tailed shearwaters (*Ardenna pacifica*) near Headstone, which are a potential prey source during high residency periods (L. Meyer, pers. obs.; Simpfendorfer et al. 2001; Meyer et al. 2010; Dicken et al. 2017). Organic waste, specifically livestock (e.g., whole animals, offal, bones, hides), has historically been discarded at Headstone (and readily consumed by tiger sharks), offering an additional food source not accessible elsewhere on the island (M. Scott, pers. obs.).

The localized use of areas around the Kingston and Cascade piers by dusky and Galapagos sharks may also be associated with incidental feeding (Meyer et al. 2021) as fishers clean their catch and dispose of waste (e.g., fish frames) at the piers. This, often daily, routine is a well‐known tourist attraction to view sharks feeding on the discards. Therefore, it is likely that these sharks frequently visit the shallow waters as part of conditioned foraging behavior. Tagging of these sharks was limited to the piers, which may have resulted in a higher propensity to revisit these locations as part of their home range. Nevertheless, the presence of mostly immature sharks was still likely associated with ontogenetic space use differences in nearshore habitat use (i.e., mature sharks primarily remaining further offshore), at least in part, driven by predator avoidance or competition (Hussey et al. 2009; Conrath and Musick 2010).

A number of tagged dusky (41%), Galapagos (44%), and sandbar (55%) sharks were rarely or never detected during the study, highlighting that we still lack a full understanding of the space use of immature sharks around Norfolk Island. There were no sex‐ or size‐associated differences between the sharks that were detected, indicating that immature individuals, independent of size, move to deeper (or other nearshore) areas not monitored. Mortality, as a result of predation, may also explain the lack of detections from some individuals given the density of predators around Norfolk Island (e.g., adult tiger sharks). Interestingly, there was no evident shift in species occupying potentially productive areas (e.g., Headstone) when tiger sharks departed seasonally. Nevertheless, white sharks ( *Carcharodon carcharias* ) may still pose a threat as they are present in the area during winter when tiger sharks are absent (M. Scott, pers. obs.). Transmitter implantation (and associated tagging) may have compounded predation risk, although tagging effects are expected to be minimal in elasmobranchs (Matley et al. 2024). In response to fishing capture, the sharks may have gained a negative association and afterwards avoided the areas. Intraspecific variation is also common within populations of elasmobranchs (McKibben and Nelson 1986; Maggs et al. 2019; Lubitz et al. 2022), where return detections may have consisted of resident individuals as opposed to more nomadic behavior in others. All species tagged are highly mobile; however, other than tiger sharks, there is limited evidence that they readily make oceanic migrations outside of continental shelves (but see Lara‐Lizardi et al. 2020) Therefore, sharks may have left Norfolk Island to exploit productive areas elsewhere (e.g., nearby seamounts) or stayed in deeper offshore areas outside the detection range of the receiver array.

### 4.1 Conclusion

Norfolk Island supports a diverse assemblage of sharks that is relatively unique, as the co‐occurrence of dusky and Galapagos sharks is extremely rare worldwide. Tiger and sandbar sharks showed limited spatial overlap with other species and evidenced seasonal migrations away from the island. The highly mobile and migratory nature of tiger sharks, predation pressures (e.g., fear effects on smaller sharks), and spatial associations to specific incidental feeding sites are key factors that may reduce competition and spatial overlap with the other species. Similarly, the preference for deeper offshore waters by sandbar sharks may restrict interactions with other species. By contrast, dusky and Galapagos sharks displayed very similar space use patterns and were present year‐round. Associated analysis has shown high rates of dietary overlap within these two species (Matley et al. in review), warranting further research into the potential impact of any resource partitioning or competition on distribution and population trends within the South Pacific Ocean, especially as they develop to adulthood. The high capture rates of immature dusky, Galapagos, and sandbar sharks raise the question of whether Norfolk Island, or certain parts of it, can be considered a nursery area. Our study lacked any description of individuals < 1 year old or young juveniles and is therefore limited to a subset of the population outside traditional definitions of nursery (Heupel et al. 2007). Nevertheless, it is evident that nearshore areas of Norfolk Island support mature and immature individuals in different ways, likely acting as a refuge to smaller species or individuals. Ultimately, genetic work is needed to characterize population connectivity with other areas in the southwestern Pacific Ocean to help determine if species at Norfolk Island are isolated. Similarly, targeting adults (for sampling) to delineate ontogenetic changes in space use, both horizontally and vertically, would be a valuable progression to further understand the ecology of sharks at Norfolk Island. Overall, the consistent use of specific areas around Norfolk Island across species further highlights the importance of offshore islands as hotspot areas fulfilling basic biological needs at both local and oceanic scales.

## Author Contributions

**Jordan K. Matley:** conceptualization (supporting), data curation (lead), formal analysis (lead), investigation (lead), methodology (equal), project administration (equal), validation (lead), visualization (lead), writing – original draft (lead), writing – review and editing (lead). **Chloe N. Roberts:** conceptualization (supporting), data curation (equal), investigation (equal), methodology (equal), project administration (equal), writing – original draft (supporting), writing – review and editing (supporting). **Thomas M. Clarke:** conceptualization (equal), data curation (equal), investigation (equal), methodology (equal), project administration (equal), validation (supporting), writing – original draft (supporting). **Lauren Meyer:** conceptualization (lead), funding acquisition (lead), investigation (equal), methodology (equal), project administration (equal), writing – original draft (supporting), writing – review and editing (supporting). **Michael P. Doane:** data curation (supporting), investigation (supporting), writing – original draft (supporting). **Elizabeth A. Dinsdale:** conceptualization (equal), funding acquisition (equal), project administration (equal), writing – original draft (supporting), writing – review and editing (supporting). **Mark Scott:** conceptualization (supporting), investigation (equal), methodology (equal), resources (supporting), writing – original draft (supporting), writing – review and editing (supporting). **Adam Barnett:** conceptualization (lead), funding acquisition (lead), investigation (equal), project administration (supporting), writing – original draft (equal), writing – review and editing (equal). **Charlie Huveneers:** conceptualization (lead), funding acquisition (lead), investigation (equal), methodology (equal), project administration (equal), validation (supporting), writing – original draft (equal), writing – review and editing (equal).

## Conflicts of Interest

The authors declare no conflicts of interest.

## Supporting information


Data S1:

## Data Availability

The data that support the findings of this study are openly available in the Australian Animal Acoustic Telemetry Database affiliated with the Integrated Marine Observing System (IMOS) at https://animaltracking.aodn.org.au/.
