# Correction to ‘Comparative Space Use of Sympatric Sharks at a Remote Island in the South Pacific Ocean’

**DOI:** 10.1002/ece3.72218

**Published:** 2025-09-22

Matley, J.K., Roberts, C.N., Clarke, T.M., Meyer, L., Doane, M.P., Dinsdale, E.A., Scott, M., Barnett, A. and Huveneers, C. (2025), Comparative Space Use of Sympatric Sharks at a Remote Island in the South Pacific Ocean. *Ecol Evol*, 15: e71534. https://doi.org/10.1002/ece3.71534

The acknowledgement of an organisation that provided equipment and support for the study was overlooked. The following sentence should have been included at the end of the *Acknowledgements* section: ‘Data were sourced from Australia's Integrated Marine Observing System (IMOS) (animaltracking.aodn.org.au)—IMOS is enabled by the National Collaborative Research Infrastructure Strategy (NCRIS). Additional equipment and support were also provided by the IMOS Animal Tracking Facility’.

We apologise for this oversight.

