# Supplementary material for: Comparative Space Use of Sympatric Sharks at a Remote Island in the South Pacific Ocean
Source: Ecol Evol. 2025 Jun 9;15(6):e71534. doi: 10.1002/ece3.71534 (PMC12146667; doi:10.1002/ece3.71534)
Supplement: Supplementary file 1 — Data S1: [file ECE3-15-e71534-s001.docx]

**Supporting Information**

Comparative space use of sympatric sharks at a small remote island in the South Pacific Ocean

Jordan K. Matley^1^*, Chloe N. Roberts^1^, Thomas M. Clarke^1^, Lauren Meyer^1,2^, Michael P. Doane^1^; Elizabeth Dinsdale^1^; Scott, Mark^3^; Barnett, Adam^4^, Charlie Huveneers^1^

^1^College of Science and Engineering, Flinders University, Adelaide, SA, 5042, Australia

^2^Georgia Aquarium, 225 Baker Street NW, Atlanta, GA, 30313, USA

^3^Norfolk Island National Park, Burnt Pine, Norfolk Island, 2899, Australia

^4^Biopixel Oceans Foundation, Cairns 4878, Australia

*Corresponding author

Table S1: GAMM output testing non-linear relationships (i.e., P-value <0.05) for monthly residency and roaming across all species. The R^2^ values for the residency and roaming models were 0.33 and 0.38, respectively.

| **Response variable** | **Smoothing level** | **Degrees of freedom (effective)** | **P-value** |
| --- | --- | --- | --- |
| **Residency** | Dusky | 0.41 | 0.298 |
|  | Galapagos | 0.51 | 0.269 |
|  | Sandbar | 2.81 | <0.001 |
|  | Tiger | 7.09 | <0.001 |
|  |  |  |  |
| **Roaming** | Dusky | 1.58 | 0.067 |
|  | Galapagos | 2.50 | <0.001 |
|  | Sandbar | <0.01 | 0.546 |
|  | Tiger | 7.13 | <0.001 |

Table S2: PERMANOVA output for dusky and Galapagos sharks based on proportional use of receiver sites.

| **Fixed effect** | **degrees of freedom** | **R^2^** | **P-value** |
| --- | --- | --- | --- |
| Species | 1 | 0.07 | 0.001 |
| Month | 11 | 0.07 | 0.001 |
| Release location | 1 | 0.29 | 0.001 |
| Size | 1 | 0.01 | 0.019 |
| Sex | 1 | 0.01 | 0.006 |
| Species*Month | 11 | 0.03 | 0.197 |
| Species*Release location | 1 | <0.01 | 0.898 |
| Species*Size | 1 | <0.01 | 0.122 |
| Species*Sex | 1 | <0.01 | 0.077 |
| Error | 198 | 0.51 |  |

Table S3: PERMANOVA output for tiger sharks based on proportional use of receiver sites.

| **Fixed effect** | **degrees of freedom** | **R^2^** | **P-value** |
| --- | --- | --- | --- |
| Month | 11 | 0.09 | 0.001 |
| Size | 1 | 0.03 | 0.001 |
| Sex | 1 | 0.04 | 0.001 |
| Month*Size | 9 | 0.01 | 0.001 |
| Month*Sex | 9 | 0.04 | 0.112 |
| Size*Sex | 1 | 0.05 | 0.992 |
| Month*Size*Sex | 7 | 0.01 | 0.973 |
| Error | 218 | 0.72 |  |


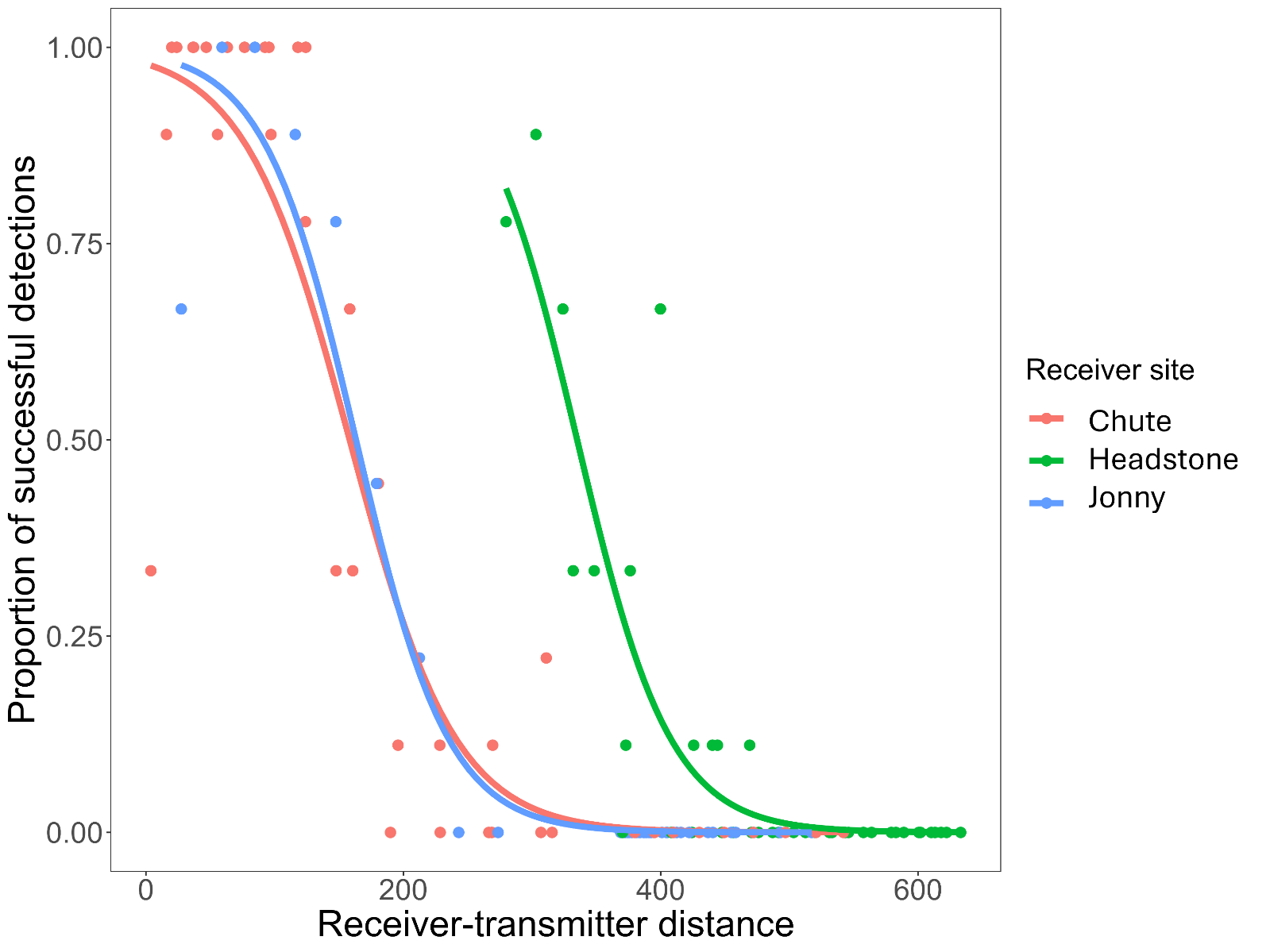


Figure S1: Detection efficiency for range testing conducted on October 2, 2023 at three sites on west side of Norfolk Island. Range testing consisted of drifting for ~20 min away from Chute and Jonny receiver sites (4 trials) with a V16 transmitter submerged (via a 5m rope) and taking GPS coordinates at regular 1-min intervals.


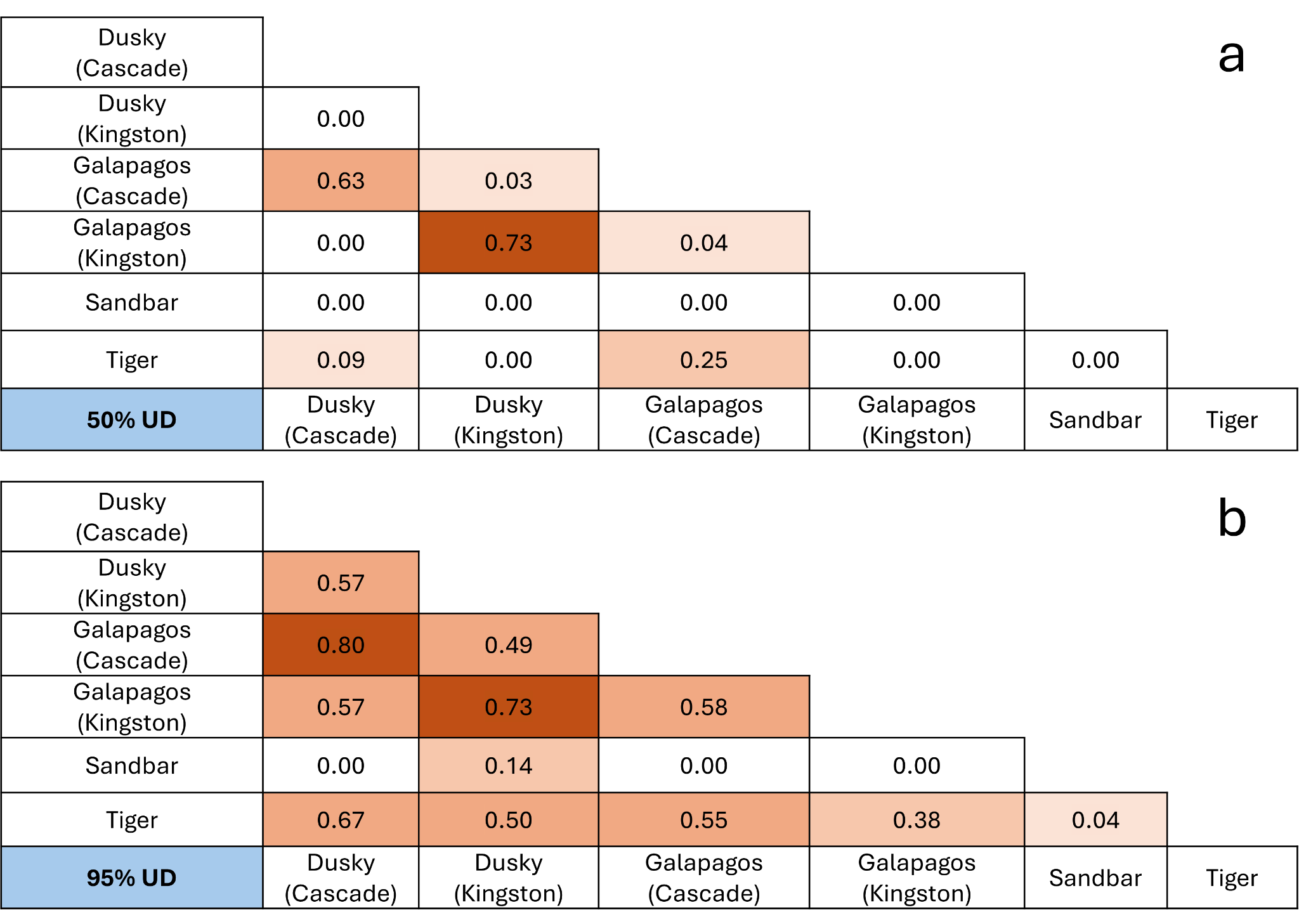


Figure S2: Activity space overlap of 50% (a) and 95% (b) kernel utilisation distributions between each species pairing including release locations for dusky and Galapagos sharks.


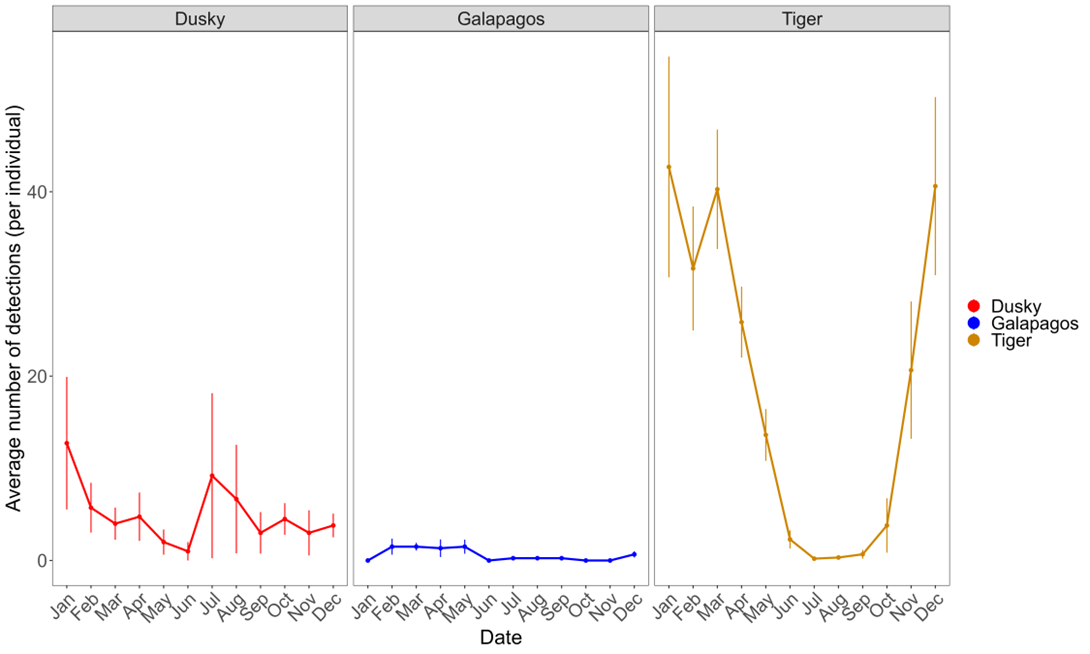


Figure S3: Mean (± standard error) number of detections (per individual) at the Headstone receiver site each month (years pooled). A total of 14 dusky, 7 Galapagos, and 38 tiger sharks were detected at the Headstone receiver site during the study period.
